# P2X_7_ regulates ependymo-radial glial cell proliferation in adult *Danio rerio* following spinal cord injury

**DOI:** 10.1242/bio.060270

**Published:** 2024-04-11

**Authors:** Eva E. Stefanova, Julian Vincent T. Dychiao, Mavis C. Chinn, Matin Borhani, Angela L. Scott

**Affiliations:** ^1^ Department of Molecular and Cellular Biology, University of Guelph, Guelph, Ontario, Canada; ^2^ Department of Pathology and Molecular Medicine, McMaster University, Hamilton, Ontario, Canada

**Keywords:** Spinal cord injury, Zebrafish, Neurogenesis, Ependymo-radial glia, Proliferation, Purines

## Abstract

In contrast to mammals, zebrafish undergo successful neural regeneration following spinal cord injury. Spinal cord ependymo-radial glia (ERG) undergo injury-induced proliferation and neuronal differentiation to replace damaged cells and restore motor function. However, the molecular cues driving these processes remain elusive. Here, we demonstrate that the evolutionarily conserved P2X_7_ receptors are widely distributed on neurons and ERG within the zebrafish spinal cord. At the protein level, the P2X_7_ receptor expressed in zebrafish is a truncated splice variant of the full-length variant found in mammals. The protein expression of this 50 kDa isoform was significantly downregulated at 7 days post-injury (dpi) but returned to basal levels at 14 dpi when compared to naïve controls. Pharmacological activation of P2X_7_ following SCI resulted in a greater number of proliferating cells around the central canal by 7 dpi but did not affect neuronal differentiation at 14 dpi. Our findings suggest that unlike in mammals, P2X_7_ signaling may not play a maladaptive role following SCI in adult zebrafish and may also work to curb the proliferative response of ERG following injury.

## Teaser

**Summary:** This study identifies, for the first time, an adaptive role for the evolutionarily conserved purinergic P2X_7_ receptor in mediating regeneration following spinal cord injury in adult zebrafish.

## INTRODUCTION

Spinal cord injury (SCI), characterized by irreversible sensory, motor, and autonomic dysfunction, is an ever-growing global health burden (Ding et al., 2022). Per year, an estimated 900,000 individuals across the world incur an SCI, significantly adding to the 20,600,000 global cases currently documented (Ding et al., 2022; Noonan et al., 2012). Although substantial progress has been made in elucidating the pathophysiology of this condition, treatment options remain extremely limited (Krueger et al., 2013; Lynch and Cahalan, 2017). This is due to the maladaptive cellular injury response within the central nervous system (CNS) that results in secondary cell death, neuroinflammation, reactive gliosis, and axonal degeneration.

In contrast to mammals, several non-mammalian vertebrate species, such as Osteichthyes, demonstrate a high degree of regenerative ability following injury to the CNS. Indeed, zebrafish (*Danio rerio*) undergo injury-induced neurogenesis following SCI and achieve functional recovery within 6–8 weeks (Becker et al., 1997; Hui et al., 2010; Reimer et al., 2008). This adaptive process is largely driven by ependymo-radial glia (ERG) lining the central canal of the spinal cord. Although the functions of ERG within the adult zebrafish spinal cord are not fully understood, in response to injury, these cells undergo a massive proliferative response and can differentiate into distinct neuronal types that are integrated into the spinal circuitry (Reimer et al., 2008). While a few signaling pathways including, but not limited to, dopamine, serotonin, and wingless-related integration site/β-catenin have been implicated in regulating this injury response, the molecular cues necessary for driving adult injury-induced neurogenesis have yet to be fully clarified (Barreiro-Iglesias et al., 2015; Briona et al., 2015; Reimer et al., 2013). Thus, identifying the signals employed by these species to restore neuron populations and locomotor function following injury may be integral to the development of neurorestorative treatments for mammals.

One evolutionarily conserved signaling pathway that has been extensively explored following SCI in mammals, but not in zebrafish, is the purinergic system (Stefanova and Scott, 2022). Nucleoside triphosphates and their metabolites regulate a plethora of cellular processes within the CNS (Burnstock and Ulrich, 2011; Gomez-Villafuertes, 2016; Oliveira et al., 2016; Ribeiro et al., 2016). These ligands have extensive receptor families, including metabotropic adenosine receptors, A_1_, A_2A_, A_2B_, A_3_; ionotropic nucleotide receptors, P2X_1–7_; and metabotropic nucleotide receptors, P2Y_1, 2, 4, 6, 11, 12, 13, 14_ (Burnstock, 2018). In the context of CNS regeneration, P2X_7_ receptors are of particular interest. These receptors function as non-selective cation channels activated by high concentrations of extracellular adenosine 5′-triphosphate (ATP). When ATP stimulation is sustained, as in the first few hours following mammalian SCI, certain splice variants of the P2X_7_ receptor may form a reversible, but cytotoxic, channel pore (Andrejew et al., 2020; De Salis et al., 2022; Di Virgilio et al., 2018; Wang et al., 2004). Other P2X_7_ splice variants have been shown to mediate proliferation and neuronal differentiation of embryonic and adult neural progenitor cells in mammals (Glaser et al., 2014; Leeson et al., 2019; Leeson et al., 2018; Tsao et al., 2013). Remarkably, while the P2X_7_ gene is present across all Metazoa, its specific properties and functions appear to differ across vertebrates (Benzaquen et al., 2019; Liang et al., 2015; López-Castejón et al., 2007; Rump et al., 2020). Given the varied, yet significant role of this receptor following mammalian SCI, in this study we focused on the P2X_7_ receptor. We aimed to uncover its potential role in injury-induced ERG cell proliferation and neuronal regeneration following SCI in adult zebrafish.

Here, we examined cellular expression of P2X_7_ receptors within the naïve and injured spinal cord, quantified temporal changes in P2X_7_ protein expression following injury, and assessed the functional significance of P2X_7_ receptor activation on injury-induced neurogenesis. Our results demonstrated that a truncated P2X_7_ variant was prominently expressed on neurons and ERG. Curiously, expression of this variant became significantly downregulated during the period of peak proliferation following SCI. Given that exogenous activation of P2X_7_ was found to promote proliferation of ERG, downregulation of P2X_7_ may act as the needed brake signal that reduces proliferation prior to differentiation of neural progenitors within the injured zebrafish spinal cord.

## RESULTS

### P2X_7_ is expressed by ependymo-radial glia and neurons within the adult zebrafish spinal cord

**Fig. 1.**
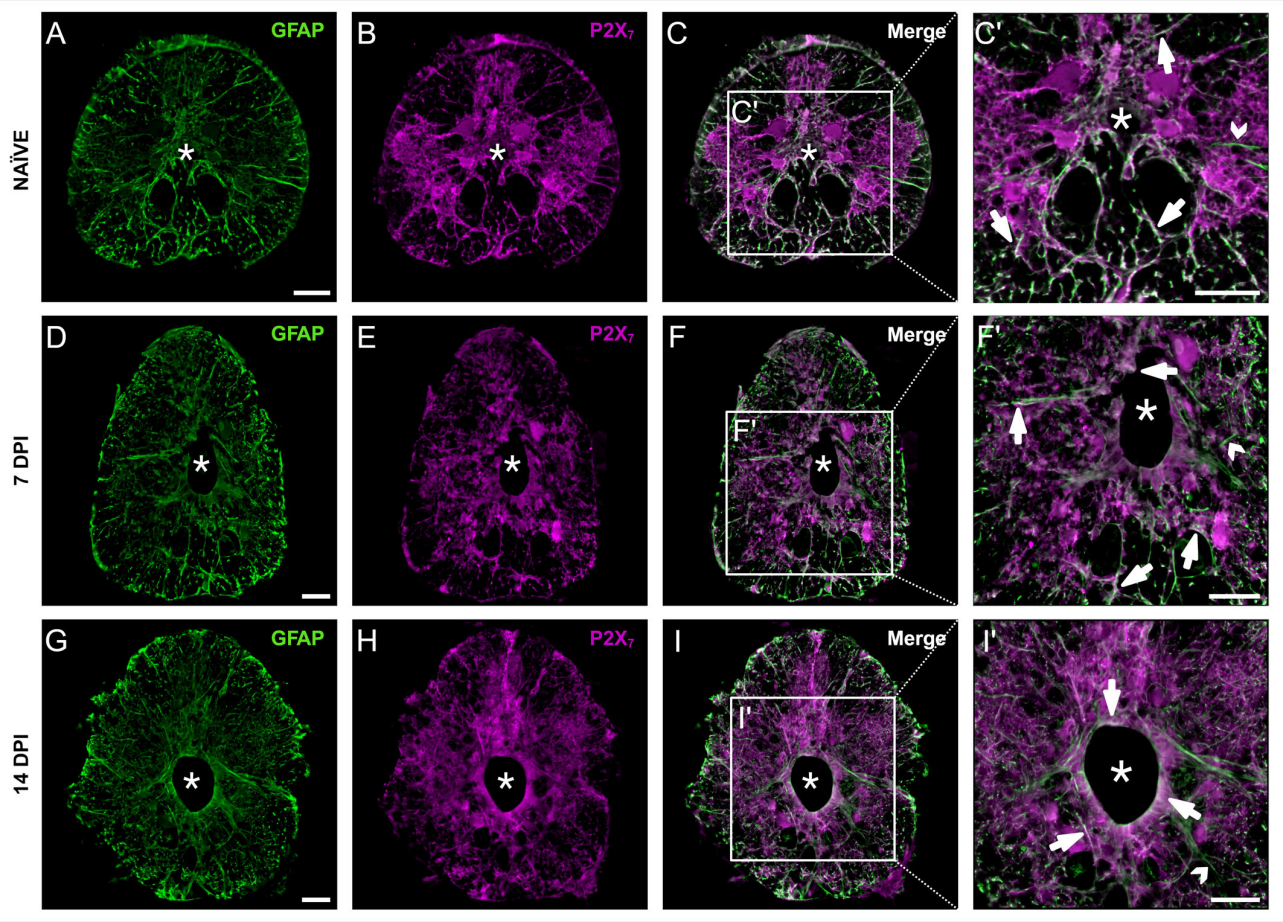
**P2X_7_ receptors were expressed by GFAP^+^ ependymo-radial glial cells.** Spinal cord cross sections are shown (dorsal is up; asterisk indicates central canal; scale bar: 50 μm). Representative images of GFAP and P2X_7_ labelling in naïve fish (A-C′), at 7 dpi (D-F′), and at 14 dpi (G-I′). Colocalization (arrows) was seen in radial processes and cell bodies (C′,F′,I′). GFAP^+^ cells that did not colocalize with P2X_7_ were also detected (open arrowheads) (C′,F′,I′). *n*=3/group. Created with BioRender.com.

In addition to mediating various homeostatic functions, ERG are essential for neural regeneration following CNS injury in zebrafish (Briona and Dorsky, 2014). The cell bodies of ERG line the central canal of the spinal cord, while their processes extend out to the pial surface. Immunohistological analyses showed that P2X_7_ colocalizes with GFAP, a marker for ERG (Becker and Becker, 2015; Lyons et al., 2003), in the thoracic spinal cord of naïve and injured (7 and 14 dpi) fish (Fig. 1). Within the naïve cord (Fig. 1A-C′), at 7 dpi (Fig. 1D-F′), and at 14 dpi (Fig. 1G-I′), colocalization of P2X_7_ and GFAP^+^ was detected in both the cell bodies and cellular processes of ERG.

**Fig. 2.**
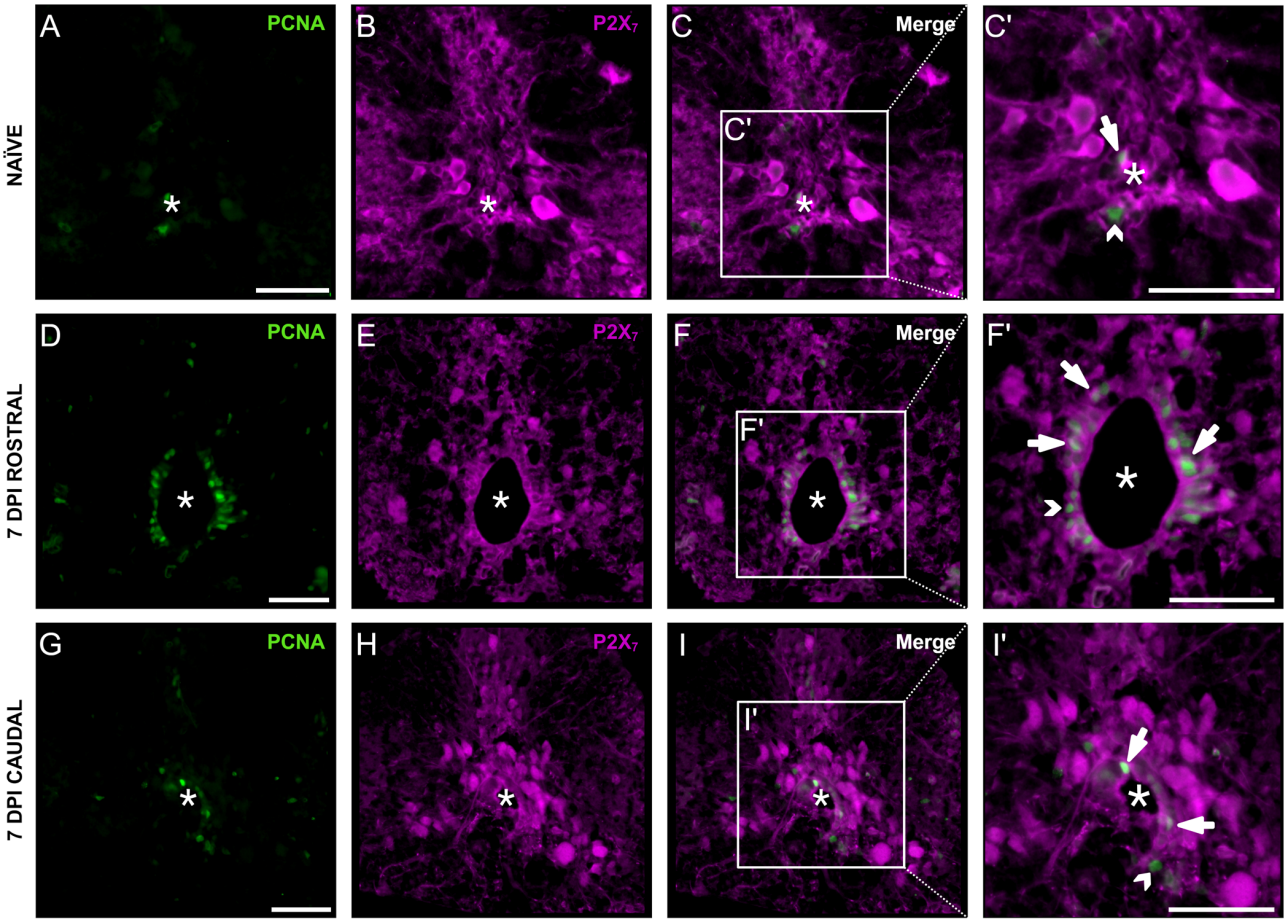
**P2X_7_ receptors were expressed by PCNA^+^ proliferating cells.** Spinal cord cross sections are shown (dorsal is up; asterisk indicates central canal; scale bar: 50 μm). Representative images of PCNA^+^ and P2X_7_ labelling in naïve fish (A-C′), at 7 dpi rostral (D-F′), and at 7 dpi caudal (G-I′). Colocalization (arrows) was seen in some cells (C′,F′,I′). PCNA^+^ cells that did not colocalize with P2X_7_ were also detected (open arrowheads) (C′,F′,I′). *n*=3/group. Created with BioRender.com.

We next examined expression of P2X_7_ on proliferating cells within the zebrafish spinal cord. During spontaneous regeneration, ERG undergo a massive proliferative response at 7 dpi (Hui et al., 2015). We found that P2X_7_ receptors are expressed by PCNA^+^ cells in the naïve zebrafish spinal cord (Fig. 2A-C′) and at 7 dpi both rostral (Fig. 2D-F′) and caudal (Fig. 2G-I′) to the lesion. Curiously, not all PCNA^+^ cells colocalized with P2X_7_. Furthermore, P2X_7_ staining was found primarily around the periphery, consistent with expression on the cellular membrane of PCNA^+^ cells (Fig. 2).

**Fig. 3.**
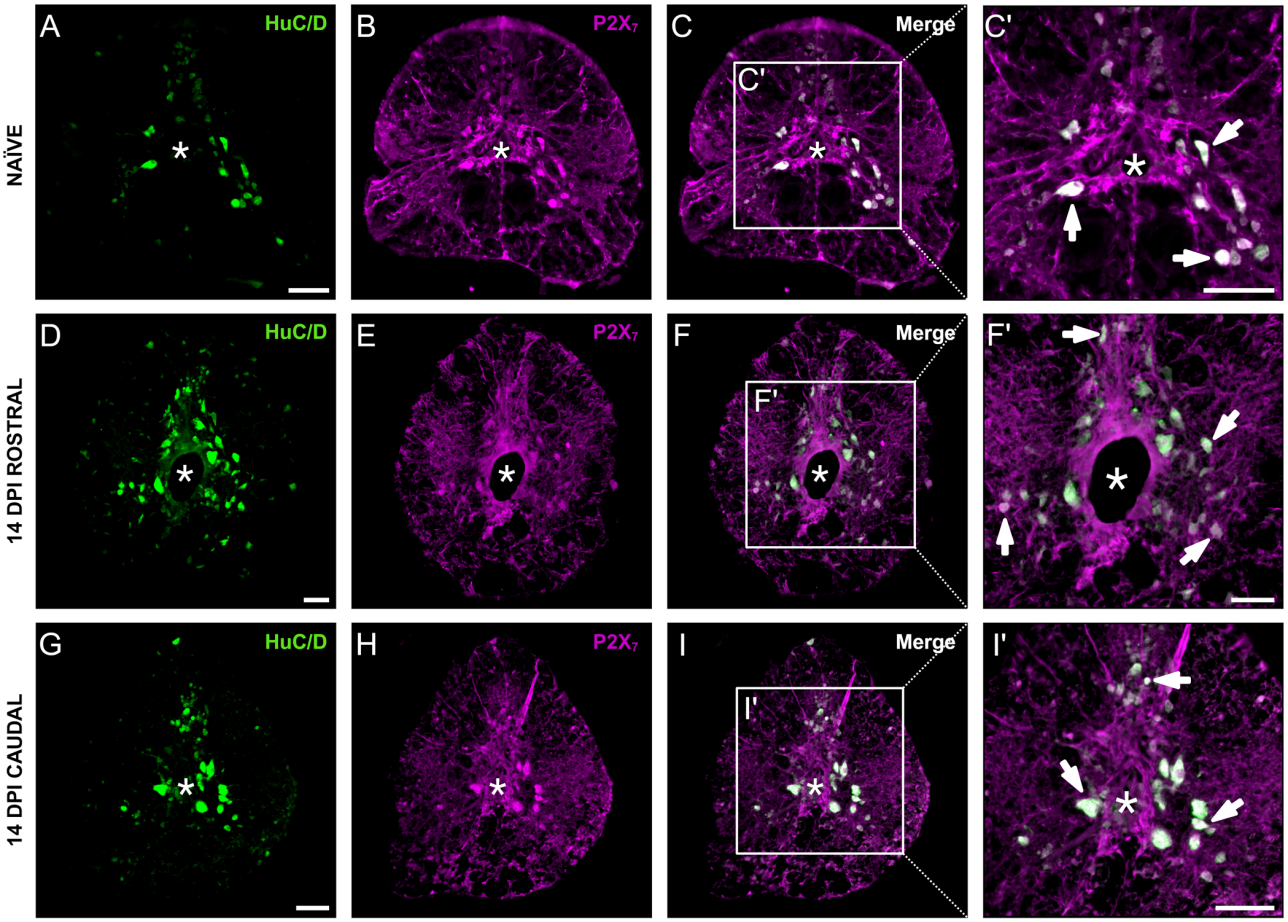
**P2X_7_ receptors were expressed by HuC/D^+^ neurons.** Spinal cord cross sections are shown (dorsal is up; asterisk indicates central canal; scale bar: 50 μm). Representative images of HuC/D^+^ and P2X_7_ labelling in naïve fish (A-C′), at 14 dpi rostral (D-F′), and at 14 dpi caudal (G-I′). Colocalization (arrows) was seen in larger mature neurons and smaller immature neurons (C′,F′,I′). *n*=3/group. Created with BioRender.com.

Lastly, we examined expression of P2X_7_ on neurons within the zebrafish spinal cord using the pan-neuronal marker HuC/D which stains both mature and immature neurons (Ghosh and Hui, 2016; Hui et al., 2010). Following SCI in adult zebrafish, peak injury-induced neurogenesis occurs at 14 dpi (Reimer et al., 2008). We found that within the adult zebrafish spinal cord, P2X_7_ receptors are expressed by HuC/D^+^ cells in naïve fish (Fig. 3A-C′) and at 14 dpi both rostral (Fig. 3D-F′) and caudal (Fig. 3G-I′) to the lesion. P2X_7_ demonstrated colocalization with small and large HuC/D^+^ cells, typically corresponding to immature and mature neurons respectively (Fig. 3).

### Differential expression of P2X_7_ following spinal cord injury in adult zebrafish

**Fig. 4.**
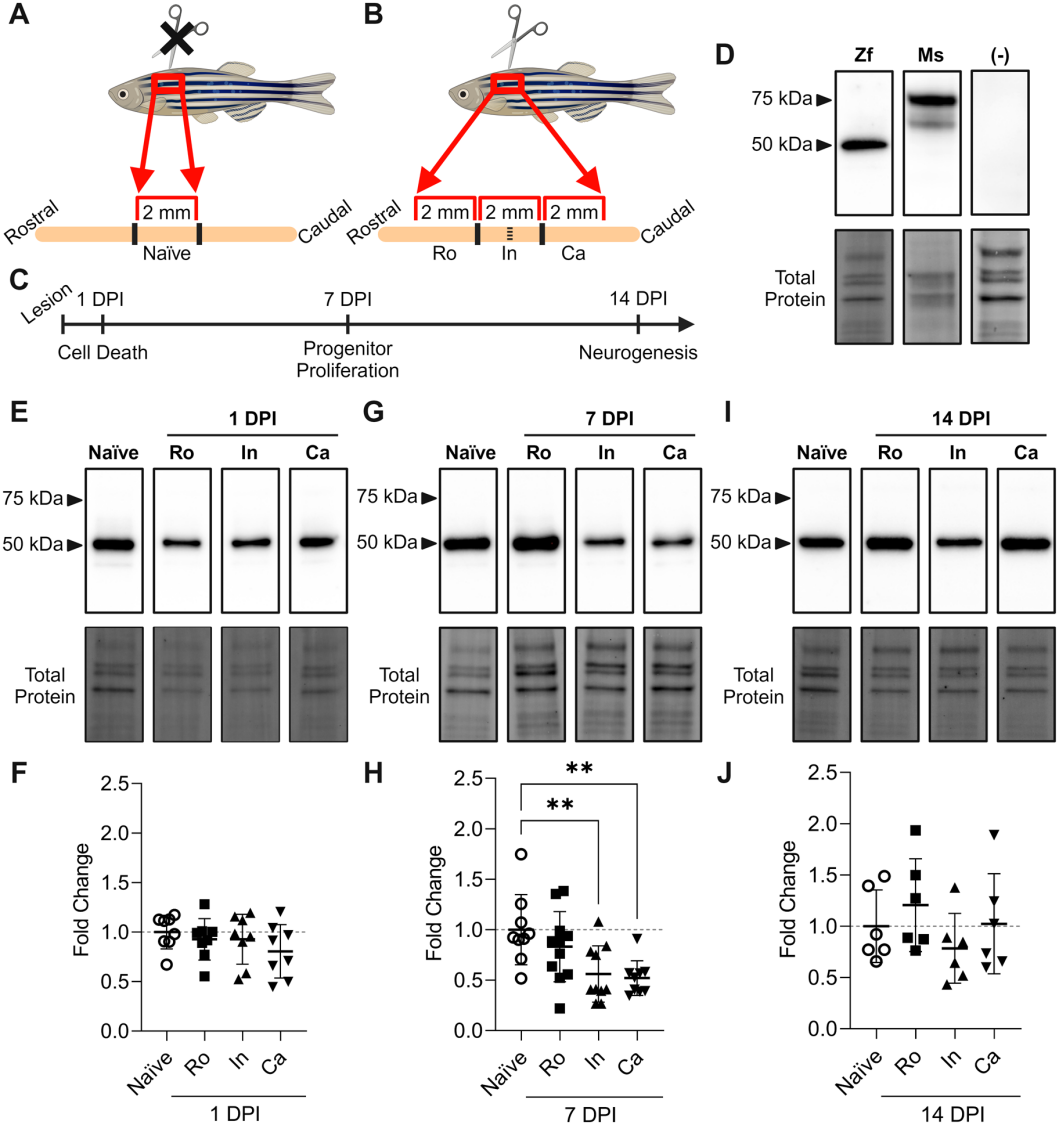
**Temporal changes in P2X_7_ protein expression following SCI in adult zebrafish.** Schematic representations of naïve and injured spinal cord areas of analysis (A-B), as well as tissue collection timeline (C). Representative western blot and corresponding total protein in naïve zebrafish (Zf) spinal cord tissue, mouse (Ms) hippocampal tissue as a positive control (+), and pre-absorption of naïve zebrafish spinal cord tissue with a blocking peptide as a negative control (-) (D). Representative western blot and corresponding total protein at 1 dpi (E), 7 dpi (G), and 14 dpi (I). Quantitative analysis of the 50 kDa isoform showed significant downregulation within the injury (In, *n*=10, one-way ANOVA with Dunnett's post hoc test, *P*=0.0079) and caudal to the lesion (Ca, *n*=9, *P*=0.0046) at 7 dpi when normalized to naïve tissue (*n*=9) (H). Protein expression of the 50 kDa isoform showed basal levels of expression at 1 dpi (F) and 14 dpi (J). Data presented as means±s.d. **, significant differences, *P*<0.01. Created with BioRender.com.

In response to mammalian SCI, protein expression of the P2X_7_ receptor is significantly upregulated by 3–7 dpi and remains elevated until at least 30 days (Fan et al., 2020; Gomez-Villafuertes et al., 2015; Kobayashi et al., 2011). To assess temporal changes in P2X_7_ protein expression following zebrafish SCI, we performed quantitative protein assays (western blotting) on spinal cord tissue collected from naïve animals and from individuals that sustained a spinal cord transection during various time periods of recovery (Fig. 4A-B). Injured tissue was collected at 1 dpi during peak secondary cell death, at 7 dpi during peak ERG cell proliferation, and at 14 dpi during peak neurogenesis (Barreiro-Iglesias et al., 2015; Hui et al., 2015; Reimer et al., 2008; Tsarouchas et al., 2018) (Fig. 4C).

Since mammals and zebrafish have highly conserved genes encoding the ‘ballast domain’, otherwise known as the extended C-terminal tail of the P2X_7_ receptor, we probed the zebrafish spinal cord with a P2X_7_ antibody targeting the full-length receptor (McCarthy et al., 2019; Rump et al., 2020). This antibody typically detects the full-length 68–85 kDa mammalian P2X_7_ receptor. We established antibody specificity for P2X_7_ using mouse brain tissue as a positive control and pre-absorption with a blocking peptide as a negative control (Carvalho et al., 2021; Leeson et al., 2018) (Fig. 4D). In zebrafish samples, we identified immunoreactive bands in the 50 kDa region; meanwhile, those in the 68–85 kDa region were either completely absent or demonstrated limited staining in just a few individuals. The molecular weight of the protein detected in zebrafish corresponded to a recognized P2X_7_ splice variant in mammals, namely P2X_7_B, that is reported to lack much of the C-terminus (Cheewatrakoolpong et al., 2005). Protein expression of the 50 kDa isoform was consistent with naïve levels at 1 dpi (Fig. 4E,F), became significantly downregulated within the injury site and caudal to the lesion at 7 dpi (Fig. 4G,H), and returned to basal levels of expression at 14 dpi (Fig. 4I,J) when compared to naïve controls.

### P2X_7_ agonism increases injury-induced ependymo-radial glial cell proliferation around the central canal

**Fig. 5.**
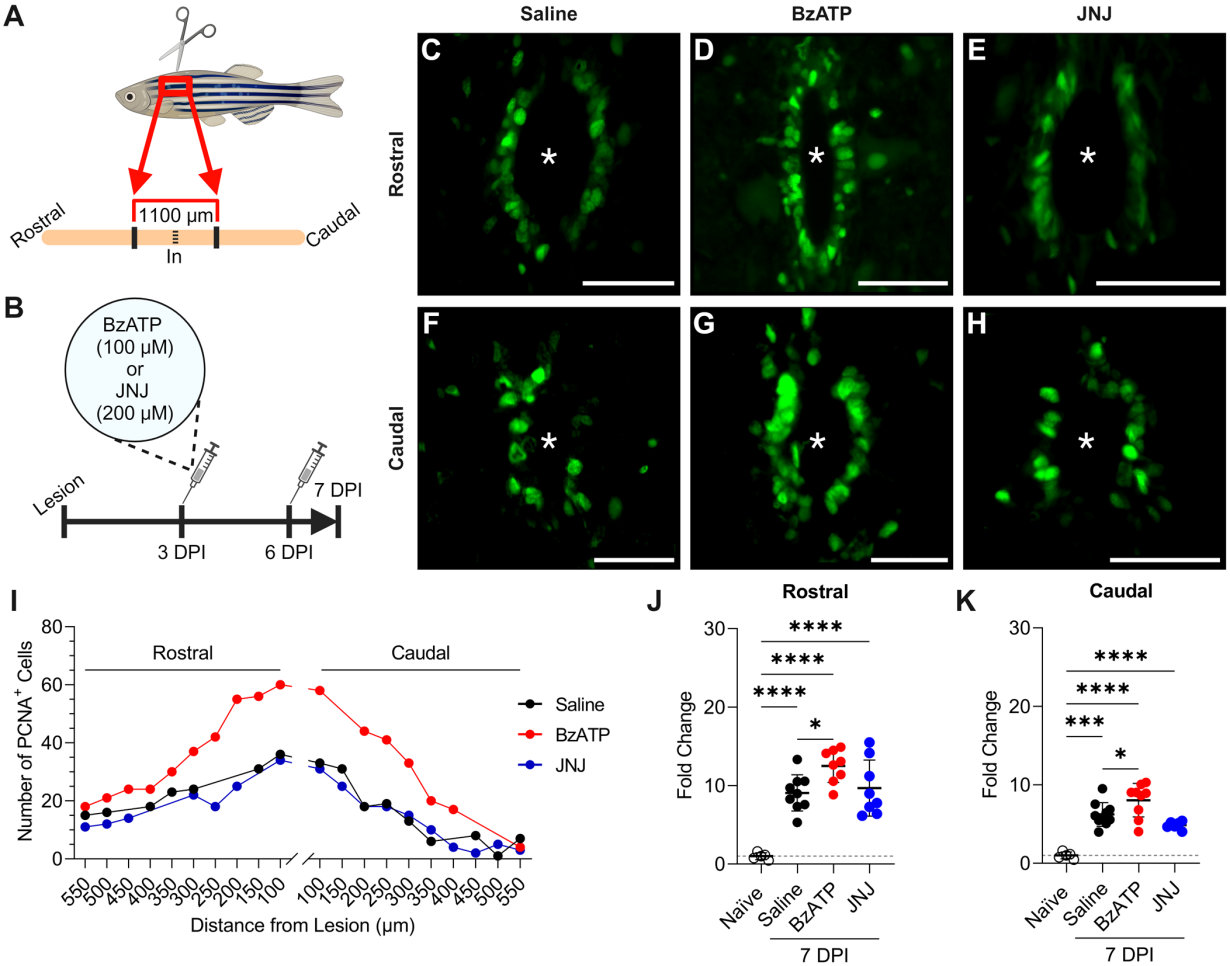
**Treatment with BzATP increased the number of PCNA^+^ cells around the central canal.** A schematic representation of the injured spinal cord area of analysis (A) as well as a timeline for the experimental condition (B). Spinal cord cross sections are shown (dorsal is up; asterisk indicates central canal; scale bar: 50 μm). Representative images of PCNA^+^ expression following treatment with saline (C,F), BzATP (D,G), and JNJ (E,H). Representative trace of PCNA^+^ expression along the length of the spinal cord in the experimental conditions (*n*=1 per treatment) (I). Quantitative analysis of summative cell counts demonstrated a significant increase in the number of PCNA^+^ expressing cells around the central canal within the first 400 μm rostral (naïve, *n*=5; saline, *n*=9; BzATP, *n*=8; JNJ, *n*=8; one-way ANOVA with Dunnett's post hoc test, *P*<0.0001) (J) and first 400 μm caudal (naïve, *n*=5; saline, *n*=10, *P*<0.0001; BzATP, *n*=9, *P*<0.0001; JNJ, *n*=6, *P*=0.0007) (K) to the lesion when normalized to naïve controls. Comparisons between saline and BzATP treated fish revealed a significant increase in the number of PCNA^+^ cells around the central canal both rostral (saline, *n*=9; BzATP, *n*=8; *P*=0.0306) (C,D,J) and caudal (saline, *n*=10; BzATP, *n*=9; *P*=0.0447) (F,G,K) to the lesion. Treatment with JNJ had no statistically significant effect on summative PCNA^+^ cell counts rostral (JNJ, *n*=8, *P*=0.8558) (E,J) or caudal (JNJ, *n*=6, *P*=0.2046) (H,K) to the lesion. Data presented as means±s.d. *, significant differences, *P*≤0.05. ***, significant differences, *P*≤0.001. ****, significant differences, *P*≤0.0001. Created with BioRender.com.

Given that ERG undergo a massive proliferative response at 7 dpi in adult zebrafish, we quantified proliferation by analyzing the number of PCNA^+^ expressing cells within the injured spinal cord at this time point (Bologna-Molina et al., 2013; Hui et al., 2015). The use of PCNA as a marker for ERG proliferation has been validated by several research groups. Specifically, it has been demonstrated that staining with bromodeoxyuridine (BrdU); a marker for cells in the S-phase of the cell cycle, and PCNA; a marker for cells in the early G_1_ and S-phases of the cell cycle, yield very similar results in the examination of ERG cell proliferation within zebrafish brain and spinal cord tissue (Grandel et al., 2006; Hui et al., 2015; Reimer et al., 2008). Consistent with previous work by Barreiro-Iglesias and colleagues (2015), we detected a significant increase in the number of PCNA^+^ expressing cells around the central canal at 7 dpi when compared to naïve controls both rostral (naïve, *n*=5; saline, *n*=9; BzATP, *n*=8; JNJ, *n*=8; one-way ANOVA with Dunnett's post hoc test, *P*<0.0001) (Fig. 5J) and caudal (naïve, *n*=5; saline, *n*=10, *P*<0.0001; BzATP, *n*=9, *P*<0.0001; JNJ, *n*=6, *P*=0.0007) (Fig. 5K) to the lesion. Quantification of PCNA^+^ cells around the central canal indicated that the number of proliferating cells decreased as distance from the lesion increased (Fig. 5I). Indeed, we noted that injury-stimulated cell proliferation around the central canal occurred primarily within the first 400 μm rostral and caudal to the lesion with relatively little effect in the distal spinal cord (Fig. 5I). Thus, for each sample (*n*), we limited our counts to spinal sections within the first 400 μm rostral and caudal to the injury site and compared the values between groups.

To directly test the involvement of P2X_7_ signaling in mediating ERG cell proliferation, we administered intraperitoneal injections of P2X_7_ agonist BzATP or P2X_7_ antagonist JNJ 47965567 at 3 and 6 dpi, and then quantified proliferation around the central canal at 7 dpi (Fig. 5B). Vehicle injections delivered in the same time intervals served as controls. We used 100 μM BzATP as this concentration specifically activates only P2X_7_ and had previously been shown to activate these receptors in larval zebrafish *in vivo* (Chang et al., 2011). We used a JNJ concentration of 200 μM based on *in vivo* studies in murine species (Bhattacharya et al., 2013; Ly et al., 2020). To test the efficacy of this concentration of JNJ in zebrafish, we performed a concentration curve analysis to test three different concentrations of the antagonist, specifically 20 μM, 200 μM, and 2 mM. The respective means of summative PCNA^+^ cell counts across the rostral spinal cord were 133.00±5.66, 191.75±70.65, and 159.50±54.45 (data not shown). All three treatment concentrations were well tolerated by adult zebrafish with no adverse effects. Given that there was no statistically significant difference between PCNA^+^ cell counts among the three groups, we selected the concentration (200 μM) previously reported to be effective *in vivo* for all subsequent experiments. Fig. 5I. When comparing vehicle to BzATP treated fish, we found a significant increase in the number of PCNA^+^ cells around the central canal both rostral (saline, *n*=9; BzATP, *n*=8; *P*=0.0306) (Fig. 5C,D,J) and caudal (saline, *n*=10; BzATP, *n*=9; *P*=0.0447) (Fig. 5F,G,K) to the lesion. Meanwhile, JNJ injections had no effect on proliferative events rostral (Fig. 5E,J) or caudal (Fig. 5H,K) to the lesion.

### P2X_7_ agonism or antagonism did not influence injury-induced neurogenesis

**Fig. 6.**
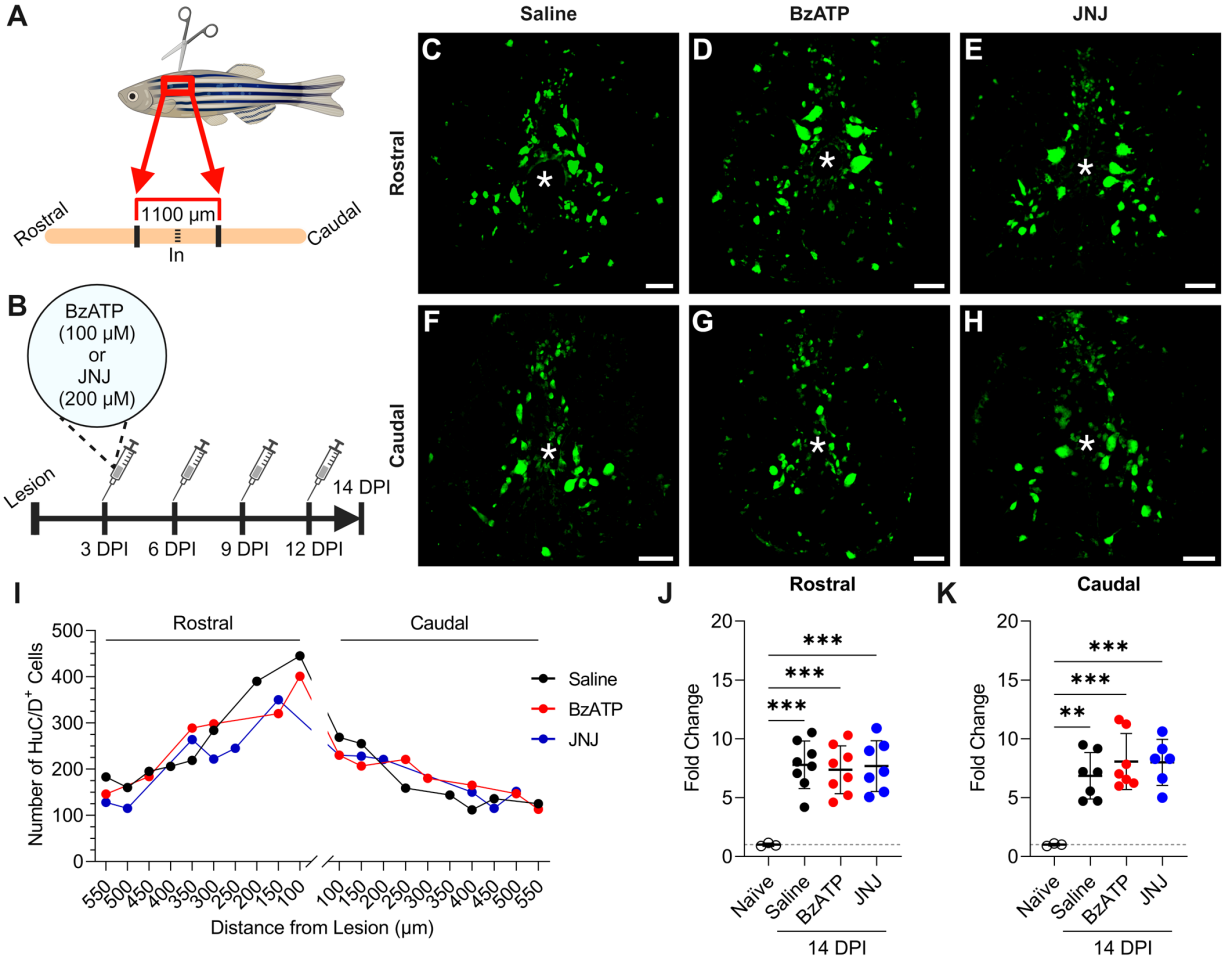
**Treatment with BzATP or JNJ had no effect on HuC/D^+^ expression within the spinal cord.** A schematic representation of the injured spinal cord area of analysis (A) as well as a timeline for the experimental condition (B). Spinal cord cross sections are shown (dorsal is up; asterisk indicates central canal; scale bar: 50 μm). Representative images of HuC/D^+^ expression following treatment with saline (C,F), BzATP (D,G), and JNJ (E,H). Representative trace of HuC/D^+^ expression along the length of the spinal cord in the experimental conditions (*n*=1 per treatment) (I). Quantitative analysis of summative cell counts demonstrated a significant increase in the total number of HuC/D^+^ cells within the first 400 μm rostral (naïve, *n*=3; saline, *n*=8; BzATP, *n*=8; JNJ, *n*=7; one-way ANOVA with Dunnett's post hoc test, *P*<0.001) (J) and caudal (naïve, *n*=3; saline, *n*=7, *P*=0.0012; BzATP, *n*=7, *P*=0.0002; JNJ, *n*=6, *P*=0.0003) (K) to the lesion. Comparisons between saline and BzATP or JNJ treated fish did not demonstrate any significant differences in the total number of HuC/D^+^ expressing cells rostral (saline, *n*=8; BzATP, *n*=8, *P*=0.8859; JNJ, *n*=7, *P*=0.9921) (J) or caudal (saline, *n*=7; BzATP, *n*=7, *P*=0.4749; JNJ, *n*=6, *P*=0.5393) (K) to the lesion. Data presented at means±s.d. **, significant differences, *P*≤0.01. ***, significant differences, *P*≤0.001. Created with BioRender.com.

Peak neurogenesis occurs at 14 dpi during spontaneous regeneration in adult zebrafish (Reimer et al., 2008). Indeed, we detected a significant increase in the total number of HuC/D^+^ cells within the spinal cord at 14 dpi when compared to naïve controls both rostral (naïve, *n*=3; saline, *n*=8; BzATP, *n*=8; JNJ, *n*=7; one-way ANOVA with Dunnett's post hoc test, *P*<0.001) (Fig. 6J) and caudal (naïve, *n*=3; saline, *n*=7, *P*=0.0012; BzATP, *n*=7, *P*=0.0002; JNJ, *n*=6, *P*=0.0003) (Fig. 6K) to the lesion. Similar findings have been obtained by groups interested in quantifying neurogenesis through mature and/or immature neuron cell counts (Barreiro-Iglesias et al., 2015; Briona and Dorsky, 2014; Ghosh and Hui, 2016; Goldshmit et al., 2018; Saraswathy et al., 2022).

To assess the involvement of P2X_7_ signaling in mediating neuronal regeneration, we administered intraperitoneal injections of P2X_7_ agonist BzATP or P2X_7_ antagonist JNJ 47965567 at 3, 6, 9, and 12 dpi, and then analyzed HuC/D^+^ expression at 14 dpi (Fig. 6B). Vehicle injections delivered in the same time frames served as controls. Quantification of HuC/D^+^ cells, which represents a compound value of both newly generated and pre-existing neurons, within the injured spinal cord indicated that the total number of neurons present decreased slightly as distance from the lesion increased (Fig. 6I). This effect was more pronounced rostral to the lesion. Again, for each sample (*n*), we limited our counts to spinal sections within the first 400 μm closest to the injury site (as above) and compared these values between treatment groups. When comparing vehicle to BzATP or JNJ treated fish, we found there were no significant differences in the total number of HuC/D^+^ expressing cells both rostral (Fig. 6C-E,J) and caudal (Fig. 6F-H,K) to the lesion.

## DISCUSSION

The P2X_7_ receptor has been implicated in various cellular functions and disease pathologies, including CNS injury. However, its role in adaptive plasticity and modulating injury-induced ERG cell proliferation and neuronal regeneration in adult zebrafish is unknown. Here, we show that the P2X_7_ receptor has widespread distribution throughout the adult zebrafish spinal cord. Remarkably, we failed to detect expression of the full-length P2X_7_ receptor, suggesting that the predominant P2X_7_ receptor isoform expressed in zebrafish differs from that expressed in murine species. Using pharmacological manipulation *in vivo*, we found that P2X_7_ receptor activation is sufficient to induce proliferation of ERG but is not a necessary mediator of either proliferation or neurogenesis in injury-induced responses.

Within the mammalian CNS, P2X_7_ receptors are expressed by immune cells, glia, and neural stem cells; however, expression by neurons appears more controversial with contradictory evidence supporting both possibilities (Fan et al., 2020; Genzen et al., 2009; Kaczmarek-Hajek et al., 2018; Khan et al., 2019; Leeson et al., 2018; Matute et al., 2007; Wang et al., 2009; Wang et al., 2004). While P2X_7_ mRNA is also expressed throughout the brain and spinal cord of larval zebrafish, thorough cellular characterization within larval or adult zebrafish CNS tissue has not yet been done (Appelbaum et al., 2007; Chang et al., 2011). In our study, we observed widespread distribution of P2X_7_ expression throughout the thoracic spinal cord of adult zebrafish with specific localization to ERG, proliferating cells around the central canal, and neurons. We found more consistent colocalization of P2X_7_ with larger HuC/D^+^ expressing neurons compared to smaller cells, suggestive of temporal changes in P2X_7_ expression over the course of neuronal maturation. Indeed, other groups have found that P2X_7_ expression changes as neural differentiation and maturation progresses in mouse embryonic stem cells and in human induced pluripotent stem cell-derived neurons (Francistiová et al., 2021; Glaser et al., 2014). These findings suggest that cellular expression of P2X_7_ may share similarities between zebrafish and mammals.

The rather diverse portfolio of roles for P2X_7_ receptors may be attributed to the existence of different splice variants that perform distinct functions (Adinolfi et al., 2018; Burnstock and Knight, 2018). Alternative splicing is highly varied across species and in terms of P2X_7_, there have been 13 splice variants identified in humans and five in mice. Of particular relevance to regeneration is the full-length protein; namely, human P2X_7_A and murine P2X_7_a, and the C-terminus truncated proteins; namely human P2X_7_B and murine P2X_7_b/P2X_7_c (Benzaquen et al., 2019; Cheewatrakoolpong et al., 2005; Masin et al., 2012; Sluyter, 2017). The full-length protein in humans and murine species retains its canonical role as an ion channel but is also capable of mediating cytotoxic cell death via pore formation (Cheewatrakoolpong et al., 2005; Di Virgilio et al., 2018; Leeson et al., 2018). Similarly, C-terminus truncated variants function as ion channels; however, unlike P2X_7_A/P2X_7_a, they demonstrate reduced channel activity and do not typically form cytotoxic pores (Cheewatrakoolpong et al., 2005; Karasawa et al., 2017; Masin et al., 2012). Prior to our study, different P2X_7_ receptor splice variants in zebrafish had not yet been identified. We detected a truncated protein matching the molecular weight of human P2X_7_B. However, the human P2X_7_ gene only shares 42% similarity with zebrafish when comparing genomic organization and chromosomal localization (Sluyter, 2017). Thus, the exact function of the zebrafish P2X_7_ protein we identified is unlikely to be identical to that in mammals because other components of the P2X_7_ receptor, like the transmembrane domains, do not share strong sequence homology between mammalian and fish species (Rump et al., 2020). These significant structural differences may account for why certain characteristic functions of mammalian P2X_7_ receptor activation, such as interleukin-1 beta (IL-1β) secretion, are not shared by zebrafish P2X_7_ receptors (López-Castejón et al., 2007). While confirmation of this potential isoform requires future analysis, our data suggests that zebrafish P2X_7_ mRNA undergoes alternative splicing to generate distinct proteins that contribute to yet unknown functions of this receptor.

Following mammalian SCI, and during pathological conditions like cancer or neurodegenerative disease, P2X_7_ receptor expression increases (Chong et al., 2010; Giuliani et al., 2014; Martin et al., 2019; Ollà et al., 2020; Pegoraro et al., 2020; Yiangou et al., 2006). Indeed, P2X_7_A mRNA and protein levels become significantly upregulated by 3–7 days post SCI and remain elevated until at least 30 days in mice (Fan et al., 2020; Gomez-Villafuertes et al., 2015; Kobayashi et al., 2011). In contrast, we showed that in adult zebrafish, P2X_7_ protein expression of the 50 kDa isoform was unchanged at 1 dpi, became downregulated at 7 dpi, and returned to basal levels of expression at 14 dpi when compared to naïve controls. In mammals, the predominant view is that early P2X_7_ upregulation potentiates secondary cell death (Peng et al., 2009; Wang et al., 2004). Thus, in zebrafish, P2X_7_ receptor expression remaining at basal levels at 1 dpi may result in neuroprotection (Hui et al., 2010; Hui et al., 2014; Ogryzko et al., 2014; Tsarouchas et al., 2018).

We hypothesize that the downregulation of P2X_7_ at 7 dpi may serve as a brake on ERG cell proliferation. In support of this, we found that pharmacological activation of P2X_7_ using BzATP increased total PCNA^+^ expression around the central canal at 7 dpi, while pharmacological inhibition with JNJ-47965567 had no effect. In agreement, multiple lines of evidence suggest that in mammals, elevated P2X_7_ receptor expression potentiates cellular proliferation in populations of highly proliferative stem or cancer cells (Francistiová et al., 2021; Giuliani et al., 2014; Pegoraro et al., 2020). Similarly, treatment of mouse embryonic stem cells with a P2X_7_ agonist increased proliferation (Glaser et al., 2014). We propose that within the zebrafish spinal cord, P2X_7_ receptor activation may serve as a potent stimulus for cellular proliferation, and under homeostatic conditions, P2X_7_ may function to maintain basal rates of ERG proliferation. To prevent uncontrolled cell growth at 7 dpi, the period of peak ERG proliferation, zebrafish downregulate protein expression of the 50 kDa P2X_7_ isoform. This downregulation does not adversely affect the rate of proliferation, suggesting that various other signaling pathways simultaneously promote ERG re-entry into the cell cycle (Barreiro-Iglesias et al., 2015; Briona et al., 2015; Reimer et al., 2009; Reimer et al., 2013).

In our study, we also detected basal levels of expression of the 50 kDa P2X_7_ isoform during the period of peak injury-induced neurogenesis (14 dpi). In response to chronic P2X_7_ receptor agonism or antagonism, we detected no statistically significant changes in the total number of HuC/D^+^ cells, indicating that P2X_7_ signaling likely does not play a principal role in neurogenesis following SCI in adult zebrafish. Within the mammalian CNS, the role of P2X_7_ in the regulation of neurogenesis is unclear. Some groups report that as neuronal differentiation progresses, P2X_7_ expression decreases (Francistiová et al., 2021; Glaser et al., 2014). Along these lines, Glaser and colleagues (2014) found that treatment of mouse embryonic stem cells with a P2X_7_ receptor agonist had no effect on neuronal differentiation, while treatment with a P2X_7_ receptor antagonist increased neuronal differentiation. In stark contrast, others suggest that P2X_7_ receptor agonism promotes neural differentiation of mouse embryonic neural progenitor cells (Tsao et al., 2013). Curiously, one group studying phenotype specification of mouse embryonal carcinoma cells found that P2X_7_ receptor activation promotes gliogenesis (Yuahasi et al., 2012). Thus, it may be possible that in our experiments, pharmacological activation of P2X_7_ pushed more ERG cells toward a glial cell fate.

An alternative explanation for the observed effect of BzATP and JNJ 47965567 on zebrafish spinal cord regeneration may be due to an indirect effect on phagocytosis. Interestingly, in the absence of extracellular ATP, P2X_7_ receptors on astrocytes or adult neural progenitor cells from rodent brains can facilitate phagocytosis *in vitro* (Leeson et al., 2018; Yamamoto et al., 2013). Phagocytosis is mediated by the closed (unbound) state of the P2X_7_ receptor. Interestingly, certain non-competitive P2X_7_ receptor antagonists like AZ10606120 block fluorescent dye uptake (via cytotoxic pore formation) without affecting P2X_7_ mediated phagocytic ability of human monocytes (Ou et al., 2018). Given that JNJ-47965567, the non-competitive P2X_7_ receptor antagonist used in this study, likely binds the same site, it seems feasible that P2X_7_ receptor inhibition by this compound similarly did not inhibit endogenous phagocytic ability (Ly et al., 2020). This is important because CNS injuries and neurodegenerative diseases are typically associated with significant cellular degeneration and cell death, which create considerable cellular debris. In cases like these, and perhaps following SCI in adult zebrafish, treatment with P2X_7_ receptor antagonists may dampen secondary cell death by inhibiting P2X_7_ mediated cytotoxic cell death. Simultaneously, these antagonists may enhance debris clearance and subsequent regeneration by promoting P2X_7_ mediated phagocytic activity.

In conclusion, our results suggest that, unlike in mammals, P2X_7_ signaling does not play a maladaptive role following SCI in adult zebrafish. Using immunohistochemical techniques, we detected P2X_7_ expression on glia and neurons. *In vivo* analysis of the effects of P2X_7_ agonism or antagonism showed that while P2X_7_ activation is sufficient to promote proliferation, it is not necessary for mediating proliferation or neurogenesis. Future studies examining the possible effects of P2X_7_ on differentiation of other cell types within the spinal cord may offer some new insights into injury-induced glial or immune cell regulation. In all, our findings suggest alternative roles of the purinergic system following CNS injury in teleost fish versus mammals.

## MATERIALS AND METHODS

### Animals

Zebrafish (*Danio rerio*) aged 3–6 months from the Tupfel Long-Fin (TL) strain were used for all experiments. Male and female fish were kept and bred in a zebrafish facility located within the Life Sciences Centre at McMaster University. All experiments were approved by the McMaster Animal Research Ethics Board (Animal Utilization Protocol 19-08-22).

### Spinal cord lesion

Prior to any surgical procedure, individual fish were acclimated to individual housing within an environmental chamber maintained at 28°C in a 14-h light cycle and fed dry food twice daily for 48 h. As previously described (Becker et al., 1997), adult fish were anesthetized by immersion in pH neutralized 0.02% aminobenzoic acid ethyl methyl ester (MS222; Sigma-Aldrich, St. Louis, MO, USA) in sterile water for ∼1 min. The anesthetized fish were then placed on a pre-wet agar plate under a dissection microscope, and a small vertical incision was made at the level of the thoracic spinal cord. The muscle layers were then blunt dissected and carefully separated with forceps, the vertebral column was exposed, and the spinal cord underwent full transection with visual confirmation of completeness. 1 μl of Vetbond tissue adhesive gel was quickly placed on the external incision. Fish were transferred to a new tank with sterile water and recovered in an environmentally controlled chamber again maintained at 28°C and set to a 14-h light cycle. Fish were not fed for 24 h post anesthesia, but regular feeding twice daily commenced afterwards and continued until the end of the experiment. Fish were housed individually and monitored twice daily to assess physical appearance (eye condition, fin and skin condition, mucus production, spinal deformities, abdomen size), clinical signs (changes in food consumption, respiratory rate, posture in water), and behaviour (hypo or hyperactivity). Fish were euthanized if they displayed grossly abnormal physical appearance, clinical signs, or behaviour. Water was changed and assessed for quality daily.

### Immunohistochemical procedures

To localize expression of select proteins, adult zebrafish spinal cord tissue was processed for histology. Zebrafish were euthanized by immersion overdose in pH neutralized 0.1% MS222 in sterile water for 5 min and immediately transcardially perfused with 200 µl saline followed by 3000 µl 4% paraformaldehyde (PFA; Sigma-Aldrich, 158127) dissolved in 0.01 M phosphate buffered saline (PBS). The vertebral column and spinal cord was then removed and placed in 4% PFA overnight before being transferred to a 20% sucrose solution (in 0.01 M PBS) for another 12–24 h. Following fixation and cryoprotection, the vertebral column was dissected to expose the injured spinal cord, the injury site was identified, and each rostral and caudal segment was isolated. From each spinal cord segment, a section of 550 µm from the injury site was embedded in Tissue Plus O.C.T. Compound Clear (ThermoFisher Scientific, Waltham, MA, USA, SGN4585) and stored in a 7×7 mm base bold (Ted Pella, Redding, CA, USA, 27147-1) that was flash frozen in liquid nitrogen and stored at −80°C. To prepare the sections for cryosectioning, the spinal cord blocks were warmed to −20°C and mounted on a sectioning stage. Frozen spinal cord sections (10 µm thick) were collected on gelatin coated slides using a Leica CM1900 cryostat. Gelatin coated slides were used to minimize detachment of sections from slides. They were pre-made by dipping frosted slides in a 40–50°C solution of 0.5% 300 bloom gelatin and 0.05% chromium potassium sulphate for 1 min. Once the liquid was drained, the slides were left to dry at 57°C for 3–4 h.

For all immunohistological markers used in this study, each set of tissue sections underwent a specific protocol for antigen retrieval prior to immunodetection. For proliferating cell nuclear antigen (PCNA), glial fibrillary acidic protein (GFAP), and P2X_7_ detection, slides were incubated in 10 mM sodium citrate and 0.05% Tween-20 (pH 6) overnight at 60°C. For detection of anti-HuC/D, slides were incubated in 50 mM Tris (pH 8) for 30 min at 85°C. Following antigen retrieval, all samples were washed in 0.01 M PBS twice for 10 min and then incubated in 1% BSA (Sigma-Aldrich) and 0.1% Triton X-100 in 0.01 M PBS for 60 min at room temperature (RT) in a humidity chamber. The blocking agent was then removed and replaced with one of the following primary antibodies diluted in 0.1% Triton X-100/0.01 M PBS: P2X_7_ (rabbit polyclonal; 1:500, Alomone Labs, APR-004, RRID: AB_2040068), GFAP (chicken polyclonal, 1:2000, ThermoFisher Scientific, PA1-10004, RRID: AB_1074620), PCNA (mouse monoclonal, 1:1000, Abcam, Cambridge, UK, ab29, RRID: AB_303394), or HuC/D (mouse monoclonal, 5 μg/ml, ThermoFisher Scientific, A-21271, RRID: AB_221448). Samples were then stored overnight at RT. The next day, samples were washed in 0.01 M PBS three times for 15 min. The slides were then incubated with one of the following secondary antibody dilutions in 0.1% Triton X-100/0.01 M PBS for 2 h at RT: donkey anti-rabbit AlexaFluor 568 (1:500, Invitrogen, Waltham, MA, USA, A10042, RRID: AB_2534017), fluorescein donkey anti-chicken (FITC; 1:200, Jackson ImmunoResearch, 703-095-155, RRID: AB_2340356), or fluorescein goat anti-mouse (FITC; 1:100, Jackson ImmunoResearch, 115-095-166, RRID: AB_2338601). Samples were washed again in 0.01 M PBS three times for 15 min. Coverslips were mounted onto slides using 2–3 drops of ProLong Gold antifade reagent with DAPI nuclear stain (Invitrogen, P36935) and stored at 4°C protected from light exposure. All spinal cord sections were imaged at 20× objective magnification using an automated stage on a Zeiss Axio Imager M2 Fluorescent microscope (Carl Zeiss, Oberkochen, Germany), Axiocam 506 camera (Carl Zeiss), and ZEN Blue (Carl Zeiss) acquisition software.

### Protein assays

Protein samples were prepared from 2 mm sections of adult zebrafish thoracic spinal cord tissue isolated from the following: (1) thoracic spinal cord tissue from naïve fish, (2) tissue within the injury site, (3) tissue adjacent to the rostral side of the injury site, and (4) tissue adjacent to the caudal side of the injury site. Tissue samples from each location of the injured zebrafish spinal cord were collected at either 1, 7, or 14 dpi.

All samples were flash frozen in liquid nitrogen and stored at −80°C before being mechanically homogenized in 20 μL of 1× Brain Extraction Buffer (25 mM HEPES pH 7.3, 150 mM KCl, 8% glycerol, 0.1% NP-40, Roche ULTRA protease inhibitor, and Roche PhoSTOP phosphatase inhibitor) using a Teflon pestle for several minutes (Reynolds et al., 2021). The homogenate was chilled on ice for 15 min, prior to centrifugation at 10,000 rpm for 10 min at 4°C. The supernatant was removed and transferred to a new microtube. Samples were chilled on ice until used in the DC protein assay (BioRad, Mississauga, Ontario), which standardized protein content for western blotting. A standard curve consisting of serial dilutions of bovine serum albumin (BSA; Sigma-Aldrich, A-7906) was prepared. The standards and samples were prepared per manufacturer's instructions (Bio-Rad). Plates were gently shaken for 15 min prior to spectrophotometer (xMark Microplate Absorbance Spectrophotometer, Bio-Rad) readings taken at 750 nm. Samples were flash frozen in liquid nitrogen and returned to storage at −80°C.

Protein samples (10 μg) were loaded on precast polyacrylamide TGX Stain-Free, 4–12% gradient, SDS gels (BioRad) and were separated electrophoretically at 125 V for 45–50 min. Each loaded protein sample corresponded to tissue from an individual fish (*n*). Protein extraction from adult mouse brain tissue was loaded as a positive control, while protein extraction from adult naïve zebrafish whole brain tissue was loaded as a cross-gel control. Following electrophoresis, gels were activated by UV exposure for 45 s to permit the visualization of total protein loaded within each lane. Proteins were transferred onto polyvinylidenedifluoride (PVDF; BioRad) membranes using the TransBlot Turbo system using the settings ‘turbo’>‘1 mini-gel’ (BioRad). Membranes were then imaged for total protein prior to incubation in 10 mM sodium citrate and 0.05% Tween-20 (pH 6) overnight at 60°C. The next day, membranes were incubated in 5% BSA (Sigma-Aldrich) in 1× Tris-buffered saline solution with Tween-20 for 1 h and then gently rotated for 3 h at RT in primary antibody dilution. This included anti-P2X_7_ (rabbit polyclonal; 1:500, Alomone Labs, Jerusalem, Israel, APR-004, RRID: AB_2040068). A negative control for the antibody was performed using a blocking peptide specific for the antibody binding site. This included a P2X_7_ control antigen (Alomone Labs, BLP-PR004, Q64663). Membranes were pre-incubated with the blocking peptide overnight at 4°C prior to primary antibody exposure (as above). Membranes were then incubated in secondary antibody (donkey anti-rabbit horseradish peroxidase, 1:2500, Cytiva Lifescience, Marlborough, MA, USA, NA934VS, RRID: AB_772206) for 2 h at RT, developed in enhanced chemiluminescence (BioRad) substrate for 5 min, and imaged using a ChemiDoc system (BioRad).

### Drug administration

At distinct intervals, adult zebrafish were treated with various compounds via intraperitoneal injections as described by Barreiro-Iglesias and colleagues (2015). Briefly, adult zebrafish were anesthetized by immersion in pH neutralized 0.02% MS222 in sterile water, removed, and placed on a pre-wet sponge under a dissection scope. The fish then underwent a single intraperitoneal injection of 20 µl saline containing one of the following dissolved compounds: 2'(3')-O-(4-Benzoylbenzoyl) adenosine-5'-triphosphate tri(triethylammonium) salt (BzATP; 100 μM, Abbexa, Cambridge, UK, abx076752) or JNJ 47965567 (200 μM, Tocris Bioscience, Bristol, UK, 5299). Vehicle injections of saline alone served as controls. Fish in experiments completed at 7 days received an injection at 3 and 6 dpi. For experiments completed at 14 days, zebrafish received an injection at 3, 6, 9, and 12 dpi.

### Experimental design and statistical analysis

For qualitative assessment of P2X_7_ colocalization with various cell types, sample sizes consisted of 3 fish per group. Meanwhile, for cell quantification experiments, the sample size was 3-10 fish per treatment group. For all immunohistochemical analysis, 4-6 sections encompassing either 550 µm naïve spinal cord or 550 µm rostral and 550 µm caudal to the lesion site were analyzed per fish (*n*). Quantification of cells detected in spinal cord transection images was completed using ImageJ with filters for background subtraction using settings for a rolling ball radius of 75 pixels. Manual counts of PCNA^+^ cells within a 30 µm radius of the central canal were completed for each section of tissue. Automated counts for HuC/D^+^ cells within the entire cross-section were completed for each section of tissue. HuC/D^+^ and DAPI^+^ channels were made into binary images that were then overlayed. The overlayed image was put through an automated cell counter and set to exclude any particles smaller than 20 pixels. All cell counts and data analysis were completed by an individual who was blinded to the experimental groups.

For quantitative protein analysis, the sample size ranged from 6–10 adult zebrafish for every time point. ImageLab 6.1 software (BioRad) was used to quantify the relative densitometry of bands of interest and total protein. Each protein of interest was normalized to the loading control and cross-gel control lanes to account for variation of membrane transfers. Results were expressed as fold change relative to the naïve control. Error bars are representative of standard deviation (s.d.). Statistical significance between groups was determined using one-way ANOVA and post-hoc Dunnett test for multiple comparisons.
